# Supplementary material for: Internal control beliefs shape positive affect and associated neural dynamics during outcome valuation
Source: Nat Commun. 2020 Mar 6;11:1230. doi: 10.1038/s41467-020-14800-4 (PMC7060341; doi:10.1038/s41467-020-14800-4)
Supplement: Supplementary file 1 — Supplementary Information [file 41467_2020_14800_MOESM1_ESM.pdf]

Supplementary Information

Internal control beliefs shape positive affect and associated neural dynamics during outcome valuation

Stolz, et al.

Supplementary Methods

Details on experimental task – studies 1-3

All subjects in a given study were tested in the same laboratory room and lighting conditions were held constant by lowering the window blinds and setting the dimmable electric lights to the same level. Brightness of the 3-5 brightest squares was randomly set on each trial within pre-defined limits in studies 1 & 2. In study 3, instead of creating a unique display by setting the previously described parameters randomly on each trial, a predefined pool of 100 grid stimuli was used, which was shuffled and therefore randomly assigned to each condition for each subject.

In each study, presentation of WIN and noWIN outcomes was manipulated, so that the overall probability of receiving each outcome would be fixed to 50% for every condition. In case a square was clicked that was not defined as belonging to the set of brightest squares on a given HC trial, a noWIN was presented in order to maintain credibility of the task. The predefined sequence of WIN and noWIN outcomes was designed in a way that no more than four WIN or noWIN trials appeared consecutively across conditions, and that no more than two WIN or noWIN trials appeared consecutively within conditions. Additionally, no condition appeared more than two times in a row. This was done in order to maintain an expectation of receiving a WIN (noWIN) outcome close to 50% across the course of the task.

Practice Task – studies 1-3

In all three studies, the practice task preceding the main experiment was conducted in order to minimize differences in outcome expectations between the three conditions. During the practice session, participants completed 15 trials of each condition without any feedback during the first twelve trials. Instead, subjects received manipulated feedback during the last three trials of the different conditions, indicating that they had "won" (LC), "chosen correctly" (MC), or "found the brightest square" (HC) in approximately 50% of the preceding trials. Precisely, there were three sets of feedback percentages (46, 50, 54 / 47, 50, 53 / 48, 50, 52), that were randomly assigned to the task condition based on the subject ID and the order of each set was randomized for each condition and every subject. In case a subject made too many mistakes (i.e. responded too slowly, clicked on the background, clicked a square of the incorrect color in the HC condition, or did not click on the grey square in the LC condition on more than ¼ of the completed practice trials) after the first eleven practice trials of a condition, the subject was informed about this by an on-screen message and the respective practice block was repeated. Before the start of the practice session, subjects were informed that depending on their performance in the practice blocks, they would be assigned to one of three groups gaining either 10, 15 or 20 €-cents on each correct trial of the main task and that the better they performed the more money they could gain during the main task. After the practice session, all subjects were informed that they had been assigned to the 20-cent group (irrespective of their performance).

Timing details on experimental task – study 1

On each trial, after a fixation of 3 seconds and a 1 second cue, the task was presented for a total of 4.5 seconds. 3 seconds after task onset, the cursor appeared at a random position over the squares and subjects could respond within 1.5 seconds. If subjects responded correctly, the outcome was presented for 2 seconds, otherwise a warning message followed without delay and informed subjects about their mistake (e.g. if they were too slow or had clicked on the background instead of a square). The sequence of conditions and outcome valence in each condition was predefined, such that no condition appeared more than twice in a row. Additionally, no more than four consecutive WIN or noWIN trials were presented across conditions, and no more than two repetitions of the same outcome valence appeared within conditions.

Timing details on experimental task – study 3

On each trial of study 3, following a fixation cross (jittered between 0.75 and 2.75 seconds) and a 1 second cue, the task was presented for a total of 4.5 seconds. The cursor appeared centrally on the screen 0.5 seconds after task onset and subjects could respond within 4 seconds. If subjects showed the requested response, following a 1.5 second delay the outcome was presented for 3 seconds. Otherwise a warning message followed requesting to follow the task rules. If a trial contained a rating, this was presented for 4 seconds on a similar scale as in study 2.

Description of Ratings

In study 1, ratings of both pride and happiness were administered after every outcome, using thermometer-like rating scales, ranging from 0 through 100, and initialized at 0 (see figure 1). Subjects could decide which affect to rate first and completed their rating by clicking a *done*-button. In study 2, either pride or happiness ratings were administered after a given trial, with a total of 24 ratings for each affect, that were evenly distributed across all four possible outcomes (6 for each combination of outcome valence (WIN, noWIN) and control (MC, HC)). For 20 out of the 39 subjects included in the analyses in study 2, the predefined assignment of pride and happiness ratings to a given outcome was switched. In study 3, only pride was rated after a total of 40 trials, equally distributed over all combinations of outcome valence and control. In studies 2 and 3 ratings were administered using horizontal rating bars, asking subjects how strongly they felt a given affect with regard to the preceding outcome. Scales ranged from *not at all* through *somewhat* to *very* and were initialized at *somewhat*, i.e. a neutral position.

Ratings of internal control beliefs in studies 1 and 2 were assessed using a paper-pencil 5-point Likert scale asking participants "How strongly could you influence the outcome in the different tasks?", with the different options being: "very strongly", "strongly", "neutral", "weakly", "very weakly". In study 3, two items were used to assess control beliefs, with the first item asking the same question as in studies 1 and 2. The second item asked "How strongly could you control the outcome in the different tasks?". In study 3, a horizontal visual analog ranging from "very weakly" through "somewhat" to "very strongly" was displayed after the main task, similar to the affect ratings and initialized at a neutral position (i.e. "somewhat"). In study 3, ratings from both items were averaged for each condition in order to measure internal control beliefs.

In addition to ratings measuring control beliefs, after the main task in study 3, a set of additional questions was asked separately for MC and HC that was later used to statistically control the relationship between the pride response and choice behavior (such as perceived percentages of WIN and noWIN outcomes they had received in the different conditions in the main task, see Supplementary Tables 8, 9, & 10). All questions were asked using a horizontal visual analog scale like the one used for the affect ratings and the control ratings in study 3 and all answers were initialized at a neutral position and recoded to range from 0 to 100.

Details on staircase-algorithm – study 3

The algorithm we used to determine the potential monetary gains of the choice options on each trial followed the logic of an adaptive staircase algorithm for the detection of perceptual detection thresholds as used in psychophysics[1]. Specifically, on each trial, one of the two choice options was selected as the reference and assigned a value of 1€, while the other option was assigned another value that was either higher or lower than the reference value. E.g., if this comparison value was higher than the reference value on a given trial and the participant picked the higher value, the comparison value was decreased by a predefined fraction in the following trials until the participant changed her decision and chose the option with the reference value.

The task was programmed to contain four phases of 24 trials each that were not explicitly conveyed to the participants. In the first phase, only comparison values were used that started with a step size of 17 cents (i.e. comparison values starting at 1.51€ or 0.49€). In the second phase, two thirds

of the trials belonged to these sets, while one third were trials starting with comparison values of 1.99€ or 0.01€ (i.e. step sizes of 33 cents). In the third phase of the task, two thirds of the trials belonged to the comparison sets with a step size of 33 cents and all of the trials in the fourth phase belonged to this set.

If on a given trial the comparison value (e.g. HC = 1.99€) was higher than the reference value (e.g. MC = 1€) and the subject picked the choice option with the (higher) comparison value, the comparison value was decreased by the current step size (e.g. 33 cents) in the following trials of the same comparison set until the subject changed her decision and picked the option with the reference value. As an example, the MC condition could be defined as the reference (1 €), while the initial comparison value of HC could be 1.99 €. Choosing HC for 1.99 € would lead to a decrease of the comparison value to 1.66 € on the next trial of this comparison set. The comparison value would be decreasing as long as HC is preferred over MC. At some point, e.g. when HC has a value of .67 €, the subject might prefer MC (having a value of 1€). Now, the comparison value is *increased* until the subject's decision changes a second time, and the comparison value starts to decrease again, this time by a smaller fraction than before (previous step size/1.25), therefore iteratively approximating the point at which the subjective value of both options are subjectively equal for this participant. On each trial, a random amount between -5 and 5 cents was added to the values in order to decrease monotony of the task. The highest offered value was 1.99€ and the lowest was 0.01€.

In each phase, 2 trials were randomly selected to be actually played by the subject (being HC or MC, depending on the subject's choice), without showing an outcome. Subjects were informed that in the end, they would receive the amount gained from those trials on which they succeeded (i.e. found the brightest square in HC; selected the correct color in MC). However, they truly received 50% of the amounts they chose and played for during the decision task.

Statistical Analyses

Corrections of *p*-values for multiple testing in R[2] were performed using the p.adjust() function from the stats package[2]. Comparisons of correlations (e.g. between affective responses and PPI effects in study 2) were performed using the cocor package[3]. Partial rank correlations in were computed using the function pcor.test() from the ppcor package[4].

Manipulation Checks

In order to verify that subjects could not reliably detect the brightest square, we computed how many times in the entire experiment the brightest square of a given color would be found given a subject performed at chance level. For instance, if in a given trial three squares were defined to be the brightest squares, there was a chance of 1/3 to find the brightest square. We computed the level of chance performance for each subject individually, accounting for the fact that by chance, the number of trials in which 3, 4, or 5 squares were the brightest could vary between subjects (due to the design of the experimental task). We performed paired t-tests in each study, comparing the number of times each subject found the brightest square with the level of chance performance. These analyses indicated, that in no study performance exceeded chance level. In fact, in every study, subjects performed significantly worse than chance (study 1: $t(39) = -33.931$, $p < .001$; study 2: $t(38) = -2.311$, $p = .026$; study 3: $t(49) = -2.762$, $p = .008$).

The objective rates of receiving WIN and noWIN outcomes did not deviate significantly from 50% in any study (study 1: LC: M=50.45%, SD=1.78, $t(39)$=1.601, two-sided p=.117, *MC*: M=50.26%, SD=1.29, $t(39)$=1.272, two-sided p=.211, HC: M=50.01%, SD=2.20, $t(39)$=0.030, two-sided p=.977; study 2: MC: M=50.10%, SD=1.14, $t(38)$=0.556, two-sided p=.581, HC: M=49.88%, SD=1.70, $t(38)$=-0.437, two-sided p=.664; study 3: MC: M=50.55%, SD=1.42, $t(49)$=0.275, two-sided p=.784, HC: M=50.00%, SD=1.15, $t(49)$=0.0005, two-sided p=.999), nor were there any significant differences in outcome rates between conditions (study 1: $F(2,78)$=0.572, p=.567; study 2: $t(38)$=-0.703, two-sided p=.487; study 3: $t(49)$=-0.234, two-sided p=.839).

Participants' subjective reports of the perceived percentages of WIN and noWIN outcomes did not differ from 50% in studies 1 and 2 (study 1: LC: M=51.60%, SD=12.36, $t(39)$=0.818, two-sided p=.418, MC: M=48.23%, SD=6.60, $t(39)$=-1.70, two-sided p=.097, HC: M=50.70%, SD=14.78, $t(39)$=.300, two-sided p=.766; study 2: MC: M=48.13%, SD=6.37, $t(37)$=-1.808, two-sided p=.079, HC: M=52.07%, SD=10.29, $t(37)$=1.216, two-sided p=.232), nor were there any significant differences between conditions (study 1: $F(2,78)$=.982, two-sided p=.379; study 2: $t(37)$=-1.936, two-sided p=.061). Surprisingly, in study 3 subjects reported having received significantly less than 50% WIN outcomes in MC (M=46.66%, SD=11.75%, $t(49)$=-2.011, two-sided p=.049, d=-0.28, 90% CI=[-.57, -.0002]), and significantly more than 50% WIN outcomes in HC (M=54.51%, SD=12.07%, $t(49)$=2.640, p=.011, two-sided, d=0.37, 90% CI=[.09, .66]), contradicting the objective outcome rates. We therefore controlled the results of study 3 for differences between conditions regarding subjective outcome rates.

Supplemental PPI analyses

In a first follow-up analysis, we focused on whether the PPI described above was driven by WIN or noWIN outcomes. Therefore, we constructed two PPI terms for each subject by computing the interaction of the left VS time-course with the contrasts HC:WIN>MC:WIN and HC:noWIN>MC:noWIN, respectively. Aside from these two PPI terms, we included regressors modelling the two task phases, two rating phases, the two contrasts used for constructing the PPI terms, the activation time-course of the left VS, as well as six realignment parameters and their first derivatives. The first level images contrasting the WIN and noWIN PPI terms were then taken to a second-level one-sample *t*-test, to test whether there were significant connectivity differences for WIN and noWIN outcomes.

A second set of follow-up analyses tested for functional connectivity of the left VS only during HC and only during MC outcomes. For each subject, we computed the interaction of the left VS activation time-course with the contrast HC>0 and the contrast MC>0, respectively. We then set up two GLMs for each subject, including, aside from the respective PPI terms, regressors modelling the two task phases, two rating phases, the contrast used for constructing the PPI term, the activation time-course of the left VS, as well as six realignment parameters and their first derivatives. In addition, for the PPI modelling HC connectivity, the outcome phases for MC (MC:WIN, MC:noWIN) were included as two additional regressors. Vice versa, for the PPI modelling MC connectivity, the outcome phases for HC (HC:WIN, HC:noWIN) were included as two additional regressors. We then computed two second-level one-sample t-tests on the first level PPI effects (contrast images for HC PPI and MC PPI).

Rationale for Masking Procedures

We created a set of masks used for region-of-interest (ROI) analyses by performing automated meta-analyses using Neurosynth[5] for the terms self referential and value, comprising activation foci of 166 and 470 studies, respectively. Using the SPM12 image calculator[6], we created three mutually exclusive image masks, showing brain regions associated with a) the term self referential, but not value (SELF¬VALUE), b) with the term value, but not self referential (VALUE¬SELF), and c) the conjunction of both terms (SELF∩VALUE, covering regions where both meta-analyses showed overlapping effects). This rationale is in line with a recent meta-analysis showing substantial overlap between regions associated with processing of SV and self-referential processes[7]. These masks were then limited to include only clusters with 5 or more contiguous voxels. For a given contrast, we first tested whether there were significant activations within the regions covered by the masks of interest. Subsequently, we tested whether the same contrast showed significant activations on the whole-brain level in order to detect regions activated that were located outside of our a-priori masks.

Supplementary Table 1. Descriptive statistics for study samples

	n	age (*M*)	age (*SD*)	age (*range*)	female/male
Study 1	40	23.33	3.11	18-31	14/26
Study 2	39	23.31	3.87	18-37	32/7
Study 3	50	23.55	3.34	18-32	31/19
overall	129	23.41	3.41	18-37	77/52

Note. In study 1, the post-experimental interview indicated that 9 out of 49 tested participants did not believe the cover story (i.e. they informed us that they did not believe that feedback depended on their behavior but was manipulated), who were therefore excluded from further analysis. In study 2, 10 out of 49 tested participants had to be excluded due to various reasons (did not believe the cover story: 6 / excessive motion: 1 / technical problems: 3). For study 3, 6 out of 56 tested participants had to be excluded from the analysis (did not believe the cover story: 4 / could not fit choice function because subject chose only one option: 1 / already participated in fMRI study: 1). *M*=mean. *SD*=standard deviation.

Supplementary Table 2. Repeated measures ANOVA of affect ratings (study 1)

Within subjects effect	*SSs*	*df*	*Mean Square*	*F*	*p*	η_p^2
condition	2018.4	1.31	1545.60	28.84[a]	<.001	.43
error	2729.2	50.93	53.59			
outcome valence	79377.8	1.00	79377.77	60.92	<.001	.61
error	50819.7	39.00	1303.07			
affect	9904.4	1.00	9904.39	17.45	<.001	.31
error	22142.1	39.00	567.74			
condition * outcome valence	4760.4	1.37	3466.75	48.51[a]	<.001	.55
error	3826.8	53.55	71.46			
condition * affect	389.4	1.31	297.16	12.87[a]	<.001	.25
error	1180.4	51.11	23.10			
outcome * affect	9825.2	1.00	9825.19	70.10	<.001	.64
error	5466.4	39.00	140.16			
condition * outcome valence * affect	548.5	1.29	426.98	11.44[a]	<.001	.23
error	1869.5	50.10	37.32			

Note. Type III sum of squares; SSs=Sum of Squares; Results are Greenhouse-Geisser corrected; [a] Mauchly's test indicates deviation from sphericity assumption.

Supplementary Table 3. Repeated measures ANOVA of affect ratings (study 2)

Within subjects effect	*SSs*	*df*	*Mean Square*	*F*	*p*	η_p^2
condition	328.1	1	328.06	8.38	.006	.18
error	1487.8	38	39.15			
outcome valence	132177.8	1	132177.83	174.83	<.001	.82
error	28728.8	38	756.02			
affect	998.3	1	998.26	12.69	.001	.25
error	2989.2	38	78.66			
condition * outcome valence	2517.4	1	2517.41	40.23	<.001	.51
error	2377.7	38	62.57			
condition * affect	119.7	1	119.75	3.24	.080	.08
error	1404.7	38	36.97			
outcome * affect	2009.8	1	2009.83	31.52	<.001	.45
error	2423.1	38	63.76			
condition * outcome valence * affect	249.3	1	249.26	9.08	.005	.19
error	1043.6	38	27.46			

Note. Type III sum of squares; SSs=Sum of Squares.

Supplementary Table 4. Repeated measures ANOVA of pride ratings (study 3)

Within subjects effect	*SSs*	*df*	*Mean Square*	*F*	*p*	η_p^2
condition	1917	1	1916.95	18.33	<.001	.27
error	5125	49	104.59			
outcome valence	30745	1	30744.92	136.19	<.001	.74
error	11062	49	225.76			
condition * outcome valence	3171	1	3171.08	35.98	<.001	.42
error	4319	49	88.15			

Note. Type III sum of squares; SSs=Sum of Squares.

Supplementary Table 5. Reaction times and number of valid trials

study	condition	Median reaction times (ms)		Number of valid trials		Number of trials planned
		M	*SD*	*M*	*SD*	
Study 1	LC	751	89	29.10	1.11	30
	MC	778	101	29.05	0.93	30
	HC	820	90	28.56	1.19	30
Study 2	MC	1428	380	38.80	1.30	40
	HC	2041	400	38.13	1.99	40
Study 3	MC	1223	325	30.76	1.24	32
	HC	1218	322	31.54	0.73	32

Note. ms = milliseconds; *M* = mean; *SD* = standard deviation; LC = low control; MC = medium control; HC = high control.

Supplementary Table 6. Pride responses and internal control (study 3)

	W	one-sided *p*	rank-biserial correlation	90% *CI* lower	90% *CI* upper
Pride response [a]	1229.00	<.001	.93	.89	.96
Control difference HC-MC [a]	1252.00	<.001	.96	.94	.98
p(HC|HC=MC) [b]	1185.00	<.001	.86	.77	.91
monetary value of control [a]	1199.00	<.001	.88	.80	.93
point of equivalence [c]	54.00	<.001	-.92	-.95	-.86

Note. a = alternative hypothesis specifies that the population median is larger than 0. b = Alternative hypothesis specifies that population median is larger than .5. c = Alternative hypothesis specifies that population median is smaller than 0.

Supplementary Table 7. Associations between pride response and control preference

	Pride response					Control difference HC - MC				
	h.	*rho*	one-sided *p*[+]	90% *CI* lower	90% *CI* upper	h.	*rho*	one-sided *p*[+]	90% *CI* lower	90% *CI* upper
p(HC|HC=MC)	+	.473	<.001	.267	.637	+	.282	.048	.050	.485
monetary value of control	+	.299	.026	.069	.500	+	.260	.048	.027	.467
point of equivalence	-	-.180	.106	-.398	.058	-	-.239	.048	-.449	-.003

Note. h.= hypothesized direction of correlation. [+] All p-values are FDR-corrected for multiple comparisons.

Supplementary Table 8. Items regarding subjects' experience of the experimental task (study 3)

	Item
subjectively received %WIN	How often do you think that you guessed correctly / identified the brightest square in the different tasks? (in percent)
difficulty (inv.)	How easy were the different tasks for you?
effort	How much effort did you put into the different tasks?
fun	How much fun did you have doing the different tasks?
exciting	How exciting / thrilling do you think the different tasks were?
exhausting	How exhausting were the different tasks?

Supplementary Table 9. Partial rank correlations of pride response and choice preference (study 3)

Correlation of pride response with:	p(choose HC|HC=MC) [a]		Monetary value of control [b]		Point of equivalence [c]	
	rho	*p*	*rho*	*p*	*rho*	*p*
controlling for HC-MC difference in:						
subjectively received %WIN	.47	<.001	.28	.024	-.16	.130
difficulty	.49	<.001	.29	.021	-.21	.070
effort	.44	<.001	.29	.024	-.19	.102
exhausting	.51	<.001	.32	.012	-.20	.082
fun	.42	.001	.23	.055	-.11	.225
exciting	.41	.002	.25	.045	-.12	.211

Note. [a] Probability to choose HC, given equal monetary offers for HC and MC. [b] Median difference between MC and HC offer on each trial. [c] value of HC relative to MC, at which subjects are equally likely to choose HC and MC. *rho* = Spearman's *rho*. *p*-values one-sided and not corrected for multiple comparisons.

Supplementary Table 10. Statistics of control variables (study 3)

	HC		MC				
	M	*SD*	*M*	*SD*	*t*	*df*	*p*
subjectively received %WIN	54.51	12.07	46.66	11.75	5.34	49	<.001
difficulty	55.16	13.83	38.9	26.15	3.93	49	<.001
effort	81.26	12.50	45.05	28.03	9.02	49	<.001
exhausting	62.15	17.51	21.65	24.18	10.01	49	<.001
fun	62.58	17.92	36.83	18.96	8.12	49	<.001
exciting	58.13	23.14	30.45	22.82	7.07	49	<.001

Note. Uncorrected p-values. HC = high control; MC = medium control; *M* = mean; *SD* = standard deviation. Two-sided *p*-values.

Supplementary Table 11. Pearson correlations of pride and happiness ratings across subjects (study 1)

			proud						happy				
			LC		MC		HC		LC		MC		HC
			no WIN	WIN	no WIN	WIN	no WIN	WIN	no WIN	WIN	no WIN	WIN	no WIN
proud	LC	WIN	.73										
	MC	noWIN	.98	.72									
		WIN	.72	.97	.69								
	HC	noWIN	.96	.69	.97	.68							
		WIN	.62	.83	.57	.90	.55						
happy	LC	noWIN	.68	.42	.70	.36	.70	.18					
		WIN	.50	.73	.46	.79	.45	.80	.41				
	MC	noWIN	.67	.42	.69	.36	.71	.19	.99	.43			
		WIN	.47	.69	.42	.78	.41	.80	.36	.98	.37		
	HC	noWIN	.62	.39	.64	.32	.69	.14	.96	.41	.97	.35	
		WIN	.46	.67	.41	.74	.39	.82	.34	.95	.36	.96	.30

Supplementary Table 12. Pearson correlations of pride and happiness ratings across subjects (study 2)

			proud				happy		
			MC		HC		MC		HC
			no WIN	WIN	no WIN	WIN	no WIN	WIN	no WIN
proud	MC	WIN	.24						
	HC	noWIN	.88	.19					
		WIN	.05	.65	-.08				
happy	MC	noWIN	.89	.08	.91	-.17			
		WIN	-.17	.52	-.12	.66	-.28		
	HC	noWIN	.90	.22	.95	-.09	.90	-.17	
		WIN	-.34	.45	-.37	.75	-.48	.86	-.36

Supplementary Table 13. Spearman correlations of pride and happiness responses across subjects (studies 1 & 2)

		90% CI		
	Spearman's rho	lower	upper	one-sided *p*
Study 1	.42	.18	.62	.003
Study 2	.24	-.03	.48	.067

Supplementary Table 14. WIN>noWIN small-volume-corrected inside VALUE¬SELF, critical T = 3.881

set		cluster				peak					MNI coordinates (mm)		
p	*c*	*p*(FWE)	*p*(FDR)	*k*	*p*(unc)	*p*(FWE)	*p*(FDR)	*T*	*Z*	*p*(unc)	x	y	z
<.001	11	<.001	.002	235	<.001	<.001	<.001	8.18	7.24	<.001	-14	8	-10
		.002	.108	61	.039	<.001	<.001	8.14	7.21	<.001	-2	58	0
						<.001	<.001	7.18	6.51	<.001	2	56	0
						<.001	.003	5.58	5.24	<.001	4	50	-6
		<.001	.002	233	<.001	<.001	<.001	7.69	6.89	<.001	16	4	-12
		<.001	.032	108	.009	<.001	<.001	6.71	6.15	<.001	-2	44	-6
						.001	.016	5.11	4.84	<.001	-4	44	-12
						.001	.030	4.93	4.68	<.001	2	46	-12
		.041	.811	1	.811	<.001	.002	5.83	5.44	<.001	0	44	0
		.015	.613	14	.297	<.001	.003	5.68	5.32	<.001	-2	-32	38
		.017	.613	12	.334	<.001	.003	5.59	5.24	<.001	4	48	-2
						<.001	.004	5.48	5.15	<.001	2	46	2
						<.001	.005	5.40	5.08	<.001	0	44	6
		.033	.811	3	.647	.012	.269	4.31	4.14	<.001	-2	40	10
		.036	.811	2	.718	.012	.269	4.30	4.13	<.001	-10	52	-2
		.041	.811	1	.811	.019	.396	4.17	4.02	<.001	-2	48	10
		.041	.811	1	.811	.033	.659	4.01	3.87	<.001	-2	48	-14

Supplementary Table 15. WIN>noWIN small-volume-corrected inside SELF∩VALUE, critical T = 4.866

set		cluster				peak					MNI coordinates (mm)		
p	*c*	*p*(FWE)	*p*(FDR)	*k*	*p*(unc)	*p*(FWE)	*p*(FDR)	*T*	*Z*	*p*(unc)	x	y	z
<.001	3	<.001	.001	111	<.001	<.001	<.001	7.60	6.82	<.001	-2	48	-4
						<.001	.021	5.86	5.47	<.001	-6	46	-4
		<.001	.592	2	.592	<.001	.005	6.29	5.82	<.001	-6	54	-4
		<.001	.592	3	.503	<.001	.034	5.69	5.33	<.001	0	-34	36

Supplementary Table 16. WIN>noWIN wholebrain p<.05, FWE-corrected

set		cluster				peak					MNI coordinates (mm)		
p	*c*	*p*(FWE)	*p*(FDR)	*k*	*p*(unc)	*p*(FWE)	*p*(FDR)	*T*	*Z*	*p*(unc)	x	y	z
<.001	12	<.001	<.001	175	<.001	<.001	<.001	8.18	7.24	<.001	-14	8	-10
		<.001	<.001	598	<.001	<.001	<.001	8.14	7.21	<.001	-2	58	0
		<.001	<.001	1425	<.001	<.001	<.001	8.12	7.20	<.001	12	-88	2
						<.001	<.001	7.23	6.54	<.001	12	-76	-10
						<.001	.002	6.77	6.20	<.001	14	-94	22
		<.001	<.001	175	<.001	<.001	<.001	7.69	6.89	<.001	16	4	-12
		<.001	<.001	163	<.001	<.001	.018	6.15	5.70	<.001	0	-34	30
						.002	.068	5.68	5.32	<.001	-2	-32	38
		.005	.127	19	.095	.001	.039	5.90	5.50	<.001	-50	-56	-20
		<.001	.005	78	.002	.001	.039	5.87	5.47	<.001	0	-10	10
		<.001	.015	54	.009	.002	.062	5.72	5.35	<.001	0	-76	50
		.014	.321	8	.267	.006	.135	5.44	5.12	<.001	28	-40	-48
		.002	.062	30	.041	.010	.217	5.30	5.00	<.001	2	-72	34
		.019	.416	5	.381	.020	.411	5.13	4.85	<.001	-34	-78	-22
		.025	.503	3	.503	.030	.617	5.01	4.75	<.001	-2	-98	6

Supplementary Table 17. HC>MC small-volume-corrected inside SELF¬VALUE, critical T = 4.027

set		cluster				peak					MNI coordinates (mm)		
p	*c*	*p*(FWE)	*p*(FDR)	*k*	*p*(unc)	*p*(FWE)	*p*(FDR)	*T*	*Z*	*p*(unc)	x	y	z
<.001	12	<.001	.004	208	<.001	<.001	<.001	6.61	6.07	<.001	-8	-58	20
						<.001	<.001	6.55	6.02	<.001	-12	-56	14
						<.001	<.001	6.31	5.83	<.001	-6	-56	14
						<.001	<.001	6.30	5.82	<.001	-6	-60	24
		.007	.527	27	.132	<.001	<.001	6.34	5.85	<.001	-8	50	-12
		<.001	.036	108	.006	<.001	<.001	6.27	5.80	<.001	-2	38	4
		.014	.649	14	.270	<.001	.005	5.60	5.25	<.001	-6	-50	6
		.040	.798	1	.798	.001	.049	5.01	4.75	<.001	-10	46	-10
		.011	.649	17	.226	.002	.073	4.88	4.64	<.001	-48	-70	26
		.035	.798	2	.699	.003	.098	4.78	4.56	<.001	-4	46	-14
		.035	.798	2	.699	.012	.333	4.43	4.25	<.001	0	48	-14
		.032	.798	3	.626	.013	.333	4.41	4.23	<.001	8	54	24
		.035	.798	2	.699	.018	.442	4.31	4.14	<.001	-8	50	16
		.040	.798	1	.798	.026	.574	4.22	4.06	<.001	4	46	-14
		.026	.798	5	.518	.037	.747	4.12	3.97	<.001	6	50	-14

Supplementary Table 18. HC>MC small-volume-corrected inside SELF∩VALUE, critical T = 3.182

set		cluster				peak					MNI coordinates (mm)		
p	*c*	*p*(FWE)	*p*(FDR)	*k*	*p*(unc)	*p*(FWE)	*p*(FDR)	*T*	*Z*	*p*(unc)	x	y	z
.050	1	.001	.016	162	.016	<.001	<.001	6.13	5.69	<.001	-8	48	-12
						<.001	<.001	5.81	5.42	<.001	-10	50	-8
						<.001	.020	4.62	4.41	<.001	0	42	4
						<.001	.020	4.60	4.40	<.001	0	40	0
						.002	.066	4.22	4.06	<.001	-8	44	0
						.004	.118	4.00	3.86	<.001	8	50	-8
						.007	.167	3.85	3.72	<.001	-6	54	-4
						.010	.213	3.73	3.62	<.001	-8	50	0
						.039	.785	3.27	3.19	.001	-14	48	-4

Supplementary Table 19. HC>MC wholebrain p<.05, FWE-corrected

set		cluster				peak					MNI coordinates (mm)		
p	*c*	*p*(FWE)	*p*(FDR)	*k*	*p*(unc)	*p*(FWE)	*p*(FDR)	*T*	*Z*	*p*(unc)	x	y	z
<.001	13	<.001	<.001	392	<.001	<.001	<.001	7.54	6.78	<.001	2	-70	-34
						<.001	.024	6.08	5.65	<.001	12	-68	-18
						.004	.159	5.56	5.22	<.001	0	-68	-16
		<.001	<.001	499	<.001	<.001	.002	6.93	6.32	<.001	-8	-56	14
						.017	.518	5.17	4.89	<.001	-6	-48	24
		<.001	.018	59	.007	<.001	.012	6.34	5.85	<.001	-8	50	-12
		<.001	.003	107	.001	<.001	.012	6.33	5.85	<.001	10	-52	14
		<.001	.004	93	.001	<.001	.013	6.27	5.80	<.001	-2	38	4
		.006	.229	16	.123	.017	.518	5.16	4.88	<.001	-22	-48	-14
		.006	.229	16	.123	.022	.619	5.09	4.82	<.001	58	-28	26
		.022	.629	4	.435	.025	.619	5.06	4.80	<.001	12	-64	-44
		.022	.629	4	.435	.025	.619	5.06	4.79	<.001	56	-52	6
		.025	.654	3	.503	.033	.780	4.98	4.73	<.001	24	-48	-24
		.036	.719	1	.719	.042	.934	4.91	4.67	<.001	16	-60	-48
		.036	.719	1	.719	.047	.949	4.88	4.64	<.001	-48	-70	26
		.036	.719	1	.719	.048	.949	4.88	4.64	<.001	-30	-34	-18

Supplementary Table 20. 2-way conjunction small-volume-corrected inside SELF∩VALUE, critical T = 3.164

set		cluster				peak					MNI coordinates (mm)		
p	*c*	*p*(FWE)	*p*(FDR)	*k*	*p*(unc)	*p*(FWE)	*p*(FDR)	*T*	*Z*	*p*(unc)	x	y	z
.050	1	.025	.486	12	.486	.022	.435	3.45	3.36	<.001	-4	48	-10

Note. Effects listed are for the contrast [HC:WIN>HC:noWIN]∩[HC:WIN>MC:WIN]

Supplementary Table 21. 2-way conjunction inside SELF∩VALUE, p<.005, uncorrected

set		cluster				peak					MNI coordinates (mm)		
p	*c*	*p*(FWE)	*p*(FDR)	*k*	*p*(unc)	*p*(FWE)	*p*(FDR)	*T*	*Z*	*p*(unc)	x	y	z
1	2	.986	.911	80	.178	.984	.691	3.45	3.36	<.001	-4	48	-10
						1.000	.772	2.97	2.91	.002	-2	42	0
		1.000	.911	1	.911	1.000	.692	3.06	3.00	.001	0	42	4

Note. Effects listed are for the contrast [HC:WIN>HC:noWIN]∩[HC:WIN>MC:WIN]

Supplementary Table 22. Paired t-test for CUE:HC>CUE:MC, small-volume corrected inside SELF∩VALUE, critical T=4.216

set		cluster				peak					MNI coordinates (mm)		
p	*c*	*p*(FWE)	*p*(FDR)	*k*	*p*(unc)	*p*(FWE)	*p*(FDR)	*T*	*Z*	*p*(unc)	x	y	z
<.001	3	.023	.448	5	.448	.003	.354	5.26	4.53	<.001	22	12	4
		.001	.041	62	.014	.009	.354	4.85	4.25	<.001	18	4	-6
						.011	.354	4.77	4.19	<.001	22	6	-4
						.012	.354	4.76	4.19	<.001	24	10	-4
						.014	.354	4.68	4.13	<.001	8	10	-6
						.016	.354	4.65	4.11	<.001	6	14	-8
						.036	.721	4.34	3.89	<.001	18	12	0
		.005	.135	26	.090	.013	.354	4.73	4.16	<.001	-12	8	-6

Supplementary Table 23. Parametric modulation HAPPY small-volume-corrected inside VALUE¬SELF, critical T = 4.237

set		cluster				peak					MNI coordinates (mm)		
p	*c*	*p*(FWE)	*p*(FDR)	*k*	*p*(unc)	*p*(FWE)	*p*(FDR)	*T*	*Z*	*p*(unc)	x	y	z
.050	1	.028	.556	3	.556	.022	.434	4.55	4.04	<.001	16	2	-12

Supplementary Table 24. Parametric modulation HAPPY wholebrain p <.0001, uncorrected

set		cluster				peak					MNI coordinates (mm)		
p	*c*	*p*(FWE)	*p*(FDR)	*k*	*p*(unc)	*p*(FWE)	*p*(FDR)	*T*	*Z*	*p*(unc)	x	y	z
<.001	9	.009	.041	91	.005	.028	.128	5.81	4.89	<.001	8	-88	6
		.047	.105	53	.023	.177	.410	5.08	4.41	<.001	-4	60	10
						.727	.680	4.31	3.87	<.001	-14	64	10
		.201	.327	24	.109	.255	.454	4.92	4.30	<.001	6	-76	-10
		.450	.517	10	.290	.351	.454	4.77	4.19	<.001	20	-88	-14
		.335	.445	15	.198	.470	.573	4.61	4.08	<.001	-4	48	-2
		.508	.517	8	.344	.523	.584	4.55	4.04	<.001	16	2	-12
		.794	.766	1	.766	.696	.680	4.35	3.89	<.001	20	-88	34
		.651	.575	4	.511	.703	.680	4.34	3.89	<.001	14	-98	8
		.651	.575	4	.511	.754	.680	4.28	3.84	<.001	26	-90	22

Supplementary Table 25. Parametric modulation PROUD small-volume-corrected inside VALUE¬SELF, critical T = 4.175

set		cluster				peak					MNI coordinates (mm)		
p	*c*	*p*(FWE)	*p*(FDR)	*k*	*p*(unc)	*p*(FWE)	*p*(FDR)	*T*	*Z*	*p*(unc)	x	y	z
<.001	7	.009	.801	22	.175	<.001	.031	6.15	5.09	<.001	-2	56	0
		.023	.801	7	.444	.002	.159	5.34	4.58	<.001	20	6	-14
		.012	.801	16	.245	.008	.306	4.86	4.26	<.001	-2	44	-6
		.040	.801	1	.801	.031	.953	4.35	3.90	<.001	-12	2	-10
		.040	.801	1	.801	.041	.953	4.25	3.82	<.001	-16	10	-8
		.035	.801	2	.703	.043	.953	4.23	3.81	<.001	18	14	-4
		.040	.801	1	.801	.048	.953	4.19	3.78	<.001	-20	10	-6

Supplementary Table 26. Parametric modulation PROUD small-volume-corrected inside SELF¬VALUE critical T = 4.353

set		cluster				peak					MNI coordinates (mm)		
p	*c*	*p*(FWE)	*p*(FDR)	*k*	*p*(unc)	*p*(FWE)	*p*(FDR)	*T*	*Z*	*p*(unc)	x	y	z
.050	1	<.001	.003	122	.003	.001	.020	5.96	4.98	<.001	-2	56	2
						.004	.074	5.27	4.54	<.001	-2	62	10

Supplementary Table 27. Parametric modulation PROUD small-volume-corrected inside SELF∩VALUE, critical T = 3.343

set		cluster				peak					MNI coordinates (mm)		
p	*c*	*p*(FWE)	*p*(FDR)	*k*	*p*(unc)	*p*(FWE)	*p*(FDR)	*T*	*Z*	*p*(unc)	x	y	z
<.001	3	.003	.163	96	.054	.001	.036	4.83	4.24	<.001	-4	48	-6
						.001	.036	4.78	4.21	<.001	0	42	-6
		.043	.870	2	.802	.002	.045	4.58	4.06	<.001	-6	54	-4
		.046	.870	1	.870	.021	.392	3.69	3.39	<.001	-8	50	0

Supplementary Table 28. Parametric modulation PROUD wholebrain p <.0001, uncorrected

set		cluster				peak					MNI coordinates (mm)		
p	*c*	*p*(FWE)	*p*(FDR)	*k*	*p*(unc)	*p*(FWE)	*p*(FDR)	*T*	*Z*	*p*(unc)	x	y	z
.001	7	<.001	.001	269	<.001	.008	.061	6.15	5.09	<.001	-2	56	0
						.084	.159	5.27	4.54	<.001	-2	62	10
						.221	.301	4.86	4.26	<.001	-2	44	-6
		.293	.532	18	.228	.071	.159	5.34	4.58	<.001	20	6	-14
		.014	.032	102	.009	.084	.159	5.27	4.54	<.001	16	-78	-8
						.109	.167	5.16	4.47	<.001	10	-88	0
		.371	.532	13	.304	.424	.498	4.54	4.03	<.001	-20	12	-4
		.661	.710	2	.710	.584	.703	4.35	3.90	<.001	-12	2	-10
		.661	.710	2	.710	.668	.762	4.25	3.82	<.001	-16	10	-8
		.588	.710	4	.581	.687	.762	4.23	3.81	<.001	18	14	-4

Supplementary Table 29. Left VS PPI (HC>MC) small-volume-corrected inside SELF¬VALUE, critical T = 4.376

set		cluster				peak					MNI coordinates (mm)		
p	*c*	*p*(FWE)	*p*(FDR)	*k*	*p*(unc)	*p*(FWE)	*p*(FDR)	*T*	*Z*	*p*(unc)	x	y	z
.001	2	.006	.244	24	.122	.001	.079	5.64	4.76	<.001	-46	-70	38
						.003	.079	5.42	4.62	<.001	-42	-72	40
		.034	.670	2	.670	.007	.143	5.07	4.39	<.001	-38	-72	40

Supplementary Table 30. Left VS PPI (HC>MC) small-volume-corrected inside SELF∩VALUE critical T = 3.360

set		cluster				peak					MNI coordinates (mm)		
p	*c*	*p*(FWE)	*p*(FDR)	*k*	*p*(unc)	*p*(FWE)	*p*(FDR)	*T*	*Z*	*p*(unc)	x	y	z
<.001	4	.033	.863	5	.649	.010	.863	4.00	3.62	<.001	-12	50	6
		.007	.569	48	.142	.017	.863	3.80	3.47	<.001	6	48	-14
						.026	.863	3.62	3.33	<.001	0	50	-12
						.032	.863	3.54	3.27	.001	-4	54	-6
		.029	.863	7	.583	.032	.863	3.54	3.27	.001	-8	46	0
						.039	.863	3.46	3.20	.001	-12	46	2
						.044	.867	3.42	3.17	.001	-8	44	-4
		.043	.863	1	.863	.038	.863	3.48	3.21	.001	-8	50	0

Supplementary Table 31. Left VS PPI (HC>MC) wholebrain p<.001, uncorrected, k >=50

set		cluster				peak					MNI coordinates (mm)		
p	*c*	*p*(FWE)	*p*(FDR)	*k*	*p*(unc)	*p*(FWE)	*p*(FDR)	*T*	*Z*	*p*(unc)	x	y	z
<.001	7	.009	.020	329	.001	.035	.481	5.67	4.78	<.001	-44	-70	40
		<.001	<.001	3147	<.001	.171	.612	5.02	4.36	<.001	22	28	38
						.174	.612	5.01	4.35	<.001	-8	46	50
						.249	.612	4.85	4.24	<.001	-14	52	12
		.307	.407	103	.042	.698	.612	4.26	3.82	<.001	-44	24	-14
						.765	.612	4.18	3.76	<.001	-34	26	-14
		.634	.576	58	.115	.718	.612	4.23	3.80	<.001	46	-64	42
		.625	.576	59	.112	.775	.612	4.16	3.75	<.001	-14	12	6
		.422	.487	84	.062	.891	.679	3.99	3.61	<.001	0	-56	16
		.054	.083	207	.006	.914	.690	3.94	3.58	<.001	-4	-48	42
						.943	.723	3.87	3.52	<.001	4	-56	36
						.983	.740	3.71	3.40	<.001	6	-46	40

Supplementary Table 32. Right VS PPI (HC>MC) small-volume-corrected inside SELF¬VALUE, critical T = 4.373

set		cluster				peak					MNI coordinates (mm)		
p	*c*	*p*(FWE)	*p*(FDR)	*k*	*p*(unc)	*p*(FWE)	*p*(FDR)	*T*	*Z*	*p*(unc)	x	y	z
.001	2	.026	.766	4	.512	.028	.821	4.59	4.07	<.001	-18	38	46
		.039	.766	1	.766	.041	.821	4.44	3.96	<.001	0	36	32

Supplementary Table 33. Right VS PPI (HC>MC) wholebrain p<.001, uncorrected, k>=50

set		cluster				peak					MNI coordinates (mm)		
p	*c*	*p*(FWE)	*p*(FDR)	*k*	*p*(unc)	*p*(FWE)	*p*(FDR)	*T*	*Z*	*p*(unc)	x	y	z
.120	3	<.001	<.001	830	<.001	.078	.205	5.35	4.59	<.001	-4	34	34
						.117	.205	5.19	4.48	<.001	-22	38	46
						.887	.910	4.01	3.64	<.001	-6	38	44
		.295	.354	101	.037	.480	.719	4.53	4.02	<.001	-38	14	54
						.998	.910	3.54	3.27	.001	-32	14	44
						.998	.910	3.54	3.27	.001	-44	16	46
		.668	.744	53	.118	.810	.910	4.13	3.73	<.001	-36	52	12

Supplementary Table 34. Left VS PPI (HC>0) wholebrain p<.001, uncorrected, k>=50

set		cluster				peak					MNI coordinates (mm)		
p	*c*	*p*(FWE)	*p*(FDR)	*k*	*p*(unc)	*p*(FWE)	*p*(FDR)	*T*	*Z*	*p*(unc)	x	y	z
<.001	10	<.001	<.001	2973	<.001	.028	.276	5.75	4.83	<.001	-10	26	60
						.033	.276	5.68	4.79	<.001	-8	56	38
						.297	.499	4.76	4.18	<.001	4	38	26
		.134	.107	154	.017	.256	.499	4.83	4.23	<.001	-40	8	56
						.532	.527	4.44	3.95	<.001	-42	16	52
						.819	.648	4.09	3.69	<.001	-32	14	50
		.028	.028	254	.003	.262	.499	4.82	4.22	<.001	48	-64	42
						.845	.675	4.06	3.66	<.001	52	-56	34
						.970	.740	3.77	3.44	<.001	46	-60	30
		.002	.003	462	<.001	.277	.499	4.79	4.20	<.001	42	28	-6
						.379	.499	4.63	4.09	<.001	46	36	-12
						.419	.499	4.58	4.05	<.001	34	22	-16
		<.001	.001	572	<.001	.293	.499	4.76	4.18	<.001	-40	40	-6
						.385	.499	4.63	4.08	<.001	-38	34	2
						.567	.544	4.40	3.92	<.001	-44	42	-16
		.124	.107	159	.015	.343	.499	4.69	4.13	<.001	-46	-68	40
		.002	.003	466	<.001	.444	.499	4.55	4.03	<.001	-4	-4	6
						.473	.499	4.51	4.00	<.001	-10	14	6
						.596	.544	4.37	3.90	<.001	-14	4	12
		.013	.015	310	.001	.481	.499	4.50	3.99	<.001	8	-56	34
						.882	.691	3.99	3.62	<.001	0	-56	36
						.920	.702	3.92	3.56	<.001	-4	-70	56
		.657	.636	56	.125	.683	.550	4.27	3.82	<.001	-28	20	-20
		.465	.413	79	.073	.914	.702	3.93	3.57	<.001	40	-60	-26
						.976	.764	3.74	3.42	<.001	30	-66	-24

Supplementary Table 35. Left VS PPI (MC>0) wholebrain p<.001, uncorrected

set		cluster				peak					MNI coordinates (mm)		
p	*c*	*p*(FWE)	*p*(FDR)	*k*	*p*(unc)	*p*(FWE)	*p*(FDR)	*T*	*Z*	*p*(unc)	x	y	z
0.99598	3	1.000	.858	1	.858	.992	.996	3.67	3.37	<.001	-20	-30	10
		.999	.858	2	.784	1.000	.996	3.37	3.13	<.001	-40	-86	14
		1.000	.858	1	.858	1.000	.996	3.33	3.09	<.001	-26	-88	18

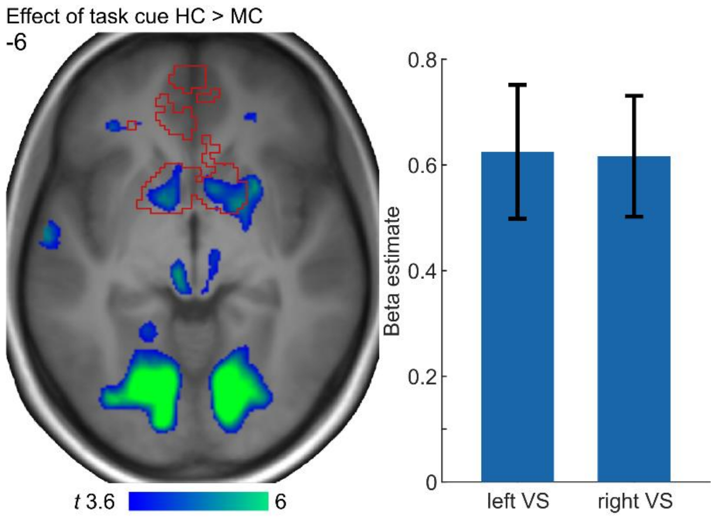

Supplementary Figure 1. Effect of cue phase. **Left** Activation map on the shows stronger activation for presenting cues signaling an upcoming HC task as compared to presenting cues indicating and upcoming MC task. Red outline shows VALUE¬SELF mask. Results are displayed at p<.0005, uncorrected. **Right** Bars show beta estimates extracted from peaks in left (x,y,z (mm): -12,8,-6) and right (18,4,-6) ventral striatum. Errorbars show +/-1 standard error of the mean. Source data are provided as a Source Data file.

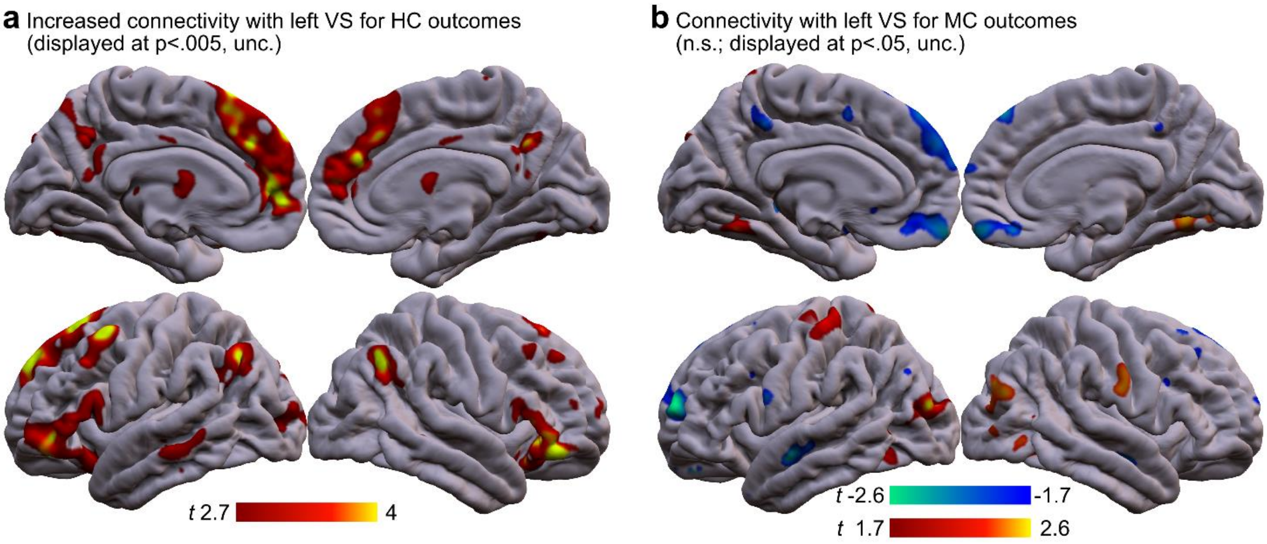

Supplementary Figure 2. Functional connectivity of the left ventral striatum (VS). **a** For HC outcomes, the PPI term was based on the interaction of the left VS time-course and the contrast HC>0. Only positive connectivity effects were found. **b** For MC outcomes, the PPI term was based on the interaction of the left VS time-course and the contrast MC>0. Connectivity effects for this PPI were generally weak, so the effects are displayed on very liberal thresholds (*p*<.05, uncorrected) for illustration purposes. Blue-green indicates negative connectivity effects, red-yellow indicates positive connectivity effects.

Supplementary References

1. Leek, M. R. Adaptive procedures in psychophysical research. *Percept. Psychophys.* **63**, 1279–1292 (2001).
2. R Core Team. R: A Language and Environment for Statistical Computing. (2018).
3. Diedenhofen, B. & Musch, J. Cocor: A comprehensive solution for the statistical comparison of correlations. *PLoS One* **10**, 1–12 (2015).
4. Kim, S. ppcor: An R Package for a Fast Calculation to Semi-partial Correlation Coefficients. *Commun. Stat. Appl. Methods* **22**, 665–674 (2015).
5. Yarkoni, T., Poldrack, R. A., Nichols, T. E., Van Essen, D. C. & Wager, T. D. Large-scale automated synthesis of human functional neuroimaging data. *Nat. Methods* **8**, 665–670 (2011).
6. Ashburner, J. *et al.* SPM12 Manual. *Functional Imaging Laboratory* 1–500 (2013). doi:10.1111/j.1365-294X.2006.02813.x
7. Acikalin, M. Y., Gorgolewski, K. J. & Poldrack, R. A. A Coordinate-Based Meta-Analysis of Overlaps in Regional Specialization and Functional Connectivity across Subjective Value and Default Mode Networks. *Front. Neurosci.* **11**, 1–11 (2017).
